# The heterogeneous ribonuclear protein C interacts with the hepatitis delta virus small antigen

**DOI:** 10.1186/1743-422X-8-358

**Published:** 2011-07-20

**Authors:** Ana Casaca, Margarida Fardilha, Edgar da Cruz e Silva, Celso Cunha

**Affiliations:** 1 Unidade de Microbiologia Médica, Centro de Malária e outras Doenças Tropicais, Instituto de Higiene e Medicina Tropical, Universidade Nova de Lisboa, Lisboa, Portugal; 2 Laboratório de Transdução de Sinais, Centro de Biologia Celular, Universidade de Aveiro, Aveiro, Portugal

**Keywords:** Hepatitis delta virus, hepatitis D small antigen, yeast two-hybrid, hnRNPC

## Abstract

**Background:**

Hepatitis delta virus (HDV) is considered to be a satellite virus of the Hepatitis B virus. The genome consists of a 1679 nt ssRNA molecule in which a single ORF was identified. This ORF codes for a unique protein, the Delta antigen (HDAg). During transcription, two forms, small (S-HDAg; p24) and large (L-HDAg; p27) of this antigen are derived as a result of an editing mechanism catalyzed by cellular adenosine deaminase 1. Despite its simplicity, little is still known about the host factors that interact with the virus RNA and antigens being to modulate virus replication.

**Methods:**

A yeast two-hybrid screening of a human liver cDNA library, using the hepatitis delta virus (HDV) small antigen (S-HDAg) as bait, was performed. Blot overlay and co-immunoprecipitation assays were used in an attempt to confirm the interaction of hnRNPC and S-HDAg. siRNA knockdown assays of hnRNPC were performed to assess the effect on HDV antigen expression.

**Results:**

Thirty known proteins were identified as S-HDAg interactors in the yeast two-hybrid screening. One of the identified proteins, hnRNPC, was found to interact with S-HDAg *in vitro *and *in vivo *in human liver cells. The interaction of the two proteins is mediated by the C-terminal half of the S-HDAg which contains a RNA-binding domain (aa 98-195). HDV RNA, S-HDAg, and hnRNPC, were also found to co-localize in the nucleus of human liver cells. Knockdown of hnRNPC mRNA using siRNAs resulted in a marked decreased expression of HDV antigens.

**Conclusions:**

S-HDAg was found to interact with human liver proteins previously assigned to different functional categories. Among those involved in nucleic acid metabolism, hnRNPC was found to interact *in vitro *and *in vivo *in human liver cells. Similar to other RNA viruses, it seems plausible that hnRNPC may also be involved in HDV replication. However, further investigation is mandatory to clarify this question.

## Background

Hepatitis delta virus (HDV) is a satellite virus of the hepatitis B virus (HBV) and the only member of the Deltagenus. The association between the two viruses is due to the fact that the HDV envelope consists of HBV surface antigens (HBsAg) which are necessary for virus propagation [1,2]. The HDV genome consists of a 1.7 Kb, circular, ssRNA molecule of negative polarity in which a single ORF was identified (reviewed in [3]). Transcription from this ORF results in the production of a 0.8 kb mRNA molecule that codes for a 195 aminoacid protein, the small delta antigen (S-HDAg). During transcription, an editing mechanism catalyzed by cellular adenosine deaminase (ADAR 1) converts an amber stop codon UAG to a tryptophan codon UGG extending this ORF by an additional 19 aminoacids resulting in the production of the large delta antigen (L-HDAg) [4]. It is generally accepted that replication of the genome occurs via a rolling-circle mechanism that involves the participation of host RNA polymerase II [5]. As a consequence, multimeric antigenomic molecules are produced which are subsequently self-cleaved and ligated at precise monomeric intervals. The newly produced antigenomes serve as templates for the synthesis of genomic RNA by a similar mechanism. Several functions have been assigned to both forms of the delta antigen and it is consensual that S-HDAg is necessary for RNA accumulation [6], and L-HDAg interacts with HBsAgs playing an important role during virus packaging [7]. However, the host factors that participate in the different steps of the HDV replication cycle interacting with both RNA and antigens are largely unknown. Recently, Cao et al reported the use of an immunoprecipitation approach followed by mass spectrometry to identify S-HDAg interactors [8]. Over 100 proteins were identified including nine RNA polymerase II subunits, transcription and splicing factors, RNA helicases, and hnRNPs.

hnRNPs are abundant nuclear proteins that belong to the large family of RNA-binding proteins containing highly conserved amino acid sequences among vertebrates [9]. These proteins associate with primary transcripts of RNA polymerase II to form hnRNP nuclear complexes. These complexes assist mRNA processing, including the stabilization of pre-mRNAs for nuclear export and translation [10,11]. The most abundant protein in hnRNP nuclear complexes is hnRNPC. Two isoforms of hnRNPC (C1 and C2) are produced by alternative splicing and hnRNPC2 was found to contain 13 additional amino acids being expressed at about one-third the level of hnRNPC1 [12]. The two isoforms form stable heterotetramers [(C1)_3_C2] that bind cooperatively to RNA [13].

It has been previously reported that several viruses interact with members of the hnRNP family. In particular, hnRNPs were shown to play important roles during replication of hepatitis C virus (HCV) [14,15], Sindbis virus [16], and vesicular stomatitis virus (VSV) [17]. Recently, hnRNPC was suggested to influence the viral replication of dengue virus by favoring survival in the host [18].

In an attempt to identify cellular factors that interact with the S-HDAg we used a yeast two-hybrid system to screen a human liver cDNA library. We were able to identify 30 known proteins, including hnRNPC. This protein was found to be expressed in 5 positive clones initially obtained and was also observed in the above mentioned study by Cao et al. [8] to interact with FLAG-S-HDAg. Here, we report that hnRNPC and S-HDAg interact *in vitro *and *in vivo*, and co-localize in human liver cells. Using plasmid constructs coding for different aminoacid sequences, in yeast two-hybrid assays, we could observe that the two proteins interact through their respective RNA binding domains. Finally, knockdown of hnRNPC in cells undergoing HDV replication, leads to a decrease in both S-HDAg and L-HDAg protein expression. Taken together, our data suggest that the S-HDAg/hnRNPC interaction here reported is specific and may be important for modulation of HDV replication.

## Methods

### Construction of plasmids

For yeast two-hybrid assays, recombinant expression plasmids were constructed using pAS2-1 (BD Biosciences). In this vector, the inserted genes are expressed in-frame with a GAL4 DNA-binding domain (GAL4BD).

To construct plasmid pAS2-1/S-HDAg, the gene coding for the S-HDAg was amplified by PCR using plasmid pSVL-AgS [19] as template and specific primers (Table 1). After digestion with *Mun*I and *Sal*I, the resulting fragment was cloned into the pAS2-1 *Eco*RI/*Sal*I sites. Three S-HDAg deletion mutants were also cloned in vector pAS2-1. The deletion mutants, encoding S-HDAg amino acids 1-62, 63-97 or 98-195 were generated by PCR amplification, using pSVL-AgS as template and the primers listed in Table 1. These plasmids were named pAS2-1/S-HDAg (1-62), pAS2-1/S-HDAg (63-97) and pAS2-1/S-HDAg (98-195).

**Table 1:** Oligonucleotides used to generate specific S-HDAg cDNA fragments by PCR

Plasmid name	Oligonucleotide	sequence (5' →3')
	
	Fwd	Rev
pAS2-1/S-HDAg	TTATCAATTGATGAGCCGGTCCGAGTCG	TTATGTCGACCTATGGAAATCCCTGGTTTCCC

pAS2-1/S-HDAg (1-62)	TTATCAATTGATGAGCCGGTCCGAGTCG	TTATGTCGACCCATCCTTATCCTTCTTTCCGAG

pAS2-1/S-HDAg (63-97)	TTATCAATTGCTGGATAAGGATGGAGAGGGG	TTATGTCGACGTCGGTGAATCCTCCCCTG

pAS2-1/S-HDAg (98-165)	TTATCAATTGAAGGAGAGGCAGGATCACCG	TTATGTCGACCTATGGAAATCCCTGGTTTCCC

pET28c/hnRNPC	TTATCAATTGGGATGGCCAGCAACGTTACC	TTATGTCGACGTGCTTAAGAGTCATCCTCGCC

For the production of recombinant proteins, the bacterial expression vectors pGEX-6P-2 (GE Healthcare) and pET28c (Novagen) were used to produce GST or His-tagged fusion proteins, respectively. To construct pGEX-6P-2/S-HDAg, for expression of GST/S-HDAg, the S-HDAg cDNA was obtained by digestion of pEGFP-C1-HDAg [20] with *Bgl*II and *Sal*I, and then inserted into the *Xho*I/*Bam*HI cloning sites of pGEX-6P-2. The plasmid for the expression of His_6_/hnRNPC, pET28c/hnRNPC, was constructed by amplifying the hnRNPC cDNA in pOTB7 (Image clone # 3354131) using the primers pET28c/hnRNPC listed in Table 1. The resulting fragment was cleaved with *Mun*I and *Sal*I, and cloned into pET28c, in the *Eco*RI/*Sal*I sites.

### Yeast two-hybrid assay

The Matchmaker GAL4 Two-hybrid system (BD Biosciences) was used to screen for proteins that interact with S-HDAg from a human liver cDNA library (BD Biosciences). Yeast two hybrid screening was performed using materials and protocols provided by the manufacturer. Briefly, *Saccharomyces cerevisiae *AH109 strain pre-transformed with bait plasmid pAS2-1/S-HDAg were mated with an Y187 strain pre-transformed with a liver cDNA library expressing fusions of GAL4 activation domain. The mating mixture was plated on synthetic medium lacking tryptophan, leucine, histidine and adenine and then assayed for α-galactosidade activity (SD/-Trp/-Leu/-Hist/-Ade/X-α-gal medium).

Plasmids isolated from positive colonies were used to transform *E. coli *DH5α cells and then sequenced. The obtained DNA sequences were then subjected to a BLAST search in the GeneBank™ database.

To verify the expression of the prey protein identified in positive clones, yeast protein extracts were prepared and analyzed by western blot. Preparation of protein samples was performed according to the Yeast Protocol Handbook from BD Biosciences. In brief, yeast cultures were grown for 2 days, harvested by centrifugation, and frozen at -80°C. The frozen cell pellets were lysed by ressupending in the same volume of warm (60°C) cracking buffer [8 M urea, 5% SDS, 40 mM Tris-HCl pH 6.8, 0.1 mM EDTA, 1% β-mercaptoethanol, 0.4 mg/mL bromophenol blue] and glass beads (425-600 μm, acid-washed, Sigma-Aldrich). The mixture was incubated at 70°C for 10 min and then vortexed for 1 min at high speed. The lysates were then cleared by centrifugation for 5 min at 14,000 rpm, and the supernatants were boiled at 100°C for 5 min. Finally, western blot analysis was performed as described below.

To confirm the detected interactions and to map the S-HDAg domains involved in the interaction with hnRNPC, yeast co-transformation assays were performed. Yeast strain AH109 was co-transformed with plasmids encoding S-HDAg deletion mutants and the prey plasmid. Growth was assayed on high stringency medium (SD/-Trp/-Leu/-Hist/-Ade/X-α-gal) to evaluate the activation reporter genes.

### Purification of recombinant proteins

Plasmids pGEX-6P-2/S-HDAg and pET28c/hnRNPC were used to transform *E. coli *BL21 and BL21-CodonPlus-(DE3)-RP competent cells, respectively. Expression of GST/S-HDAg and His/hnRNPC proteins was induced with 1 mM isopropyl-β-D-thiogalactopyranoside (IPTG) for 2 h. After centrifugation, bacterial cells were ressuspended in PBS containing 0.1 mg/mL lysozyme. Three freeze-thaw cycles were performed and then the samples were treated with DNAse I (Roche).

Purification of S-HDAg from whole bacterial protein samples was performed using a GSTrap FF 1 mL column (GE Healthcare), according to the manufacturer's instructions. Briefly extracts were loaded onto a column and extensively washed. PreScission protease (GE Healthcare) was then added to remove the GST-tag from GST/S-HDAg. The eluted fraction containg S-HDAg protein was dialyzed against PBS and quantified using a Bradford assay based kit (BioRad).

### Blot overlay assay

The blot overlay analysis was performed as previously described [21] using bacterial extracts containing the His_6_/hnRNPC recombinant protein. Increasing amounts of these extracts were separated by SDS-PAGE on a 12% gel, and transferred to a nitrocellulose membrane. The membrane was blocked with PBS containing 5% low fat milk powder and subsequently probed with 40 μg of purified recombinant S-HDAg protein in the same buffer, for 1 h at room temperature. After washing, bound S-HDAg was detected by immunoblot using a rabbit polyclonal anti-HDAg antibody (B3) [22], as described below.

### Cell culture

The human hepatocellular carcinoma cell line Huh7 (European Collection of Cell Cultures n° 01042712) and the HDV cDNA stably transfected Huh7-D12 cell line (Japanese Collection of Research Bioresources JCRB 0403) were used in this study. The Huh7-D12 cell line was obtained by stable transfection of Huh7 cells with a plasmid containing a trimer of full-length genomic HDV cDNA. The cDNA was initially obtained from a woodchuck liver infected with human serum containing HDV. Huh7-D12 cells constitutively express HDV ribonucleoproteins in a pattern consistent with ongoing HDV replication [23,24].

Both cell lines were cultured as monolayers in RPMI 1640 medium (Sigma) supplemented with 10% fetal bovine serum (Invitrogen) and grown at 37°C in a humidified atmosphere containing 5% CO_2_.

### Co-immunoprecipitation

For co-immunoprecipitation, protein extracts from Huh7-D12 or Huh7 cells were prepared. Cells were first sedimented by centrifugation and resuspended in lysis buffer [50 mM Tris-HCl (pH 7.5), 150 mM NaCl, 1% Nonidet P-40 and 10% glycerol] supplemented with complete protease inhibitor cocktail (Roche). After preclearing with protein G Dynabeads (Invitrogen), extracts were incubated with a rabbit polyclonal antibody anti-HDAg, at 4°C for 2 h. The resulting immunocomplexes were precipitated with Protein G Dynabeads (Invitrogen), extensively washed with PBS, and analyzed by western blot.

### Western blot

Western blot analysis was performed essentially as previously described [23]. Protein samples were separated on 12% SDS-PAGE gels and transferred to nitrocellulose membranes (Schleicher & Schuell). The membranes were blocked with PBS containing 5% low fat milk powder and probed with specific primary antibodies, followed by incubation with appropriate secondary antibodies conjugated with horseradish peroxidase (BioRad). Blots were developed with an enhanced chemiluminescence system (ECL, GE Healthcare). When needed, the density of the protein bands was determined using the NIH ImageJ software http://rsbweb.nih.gov/ij/.

The following primary antibodies were used: mouse monoclonal antibody against hnRNPC (Santa Cruz Biotechnology), rabbit polyclonal anti-HDAg antibody, mouse anti-His-tag monoclonal antibody (Sigma-Aldrich), and rabbit polyclonal anti-clathrin antibody (Santa Cruz Biotechnology).

### Immunofluorescence and *in situ *hybridization

Immunofluorescence and *in situ *hybridization were performed on Huh7-D12 cells, as earlier described [23].

For immunofluorescence cells, grown on coverslips, were fixed with 3.7% formaldehyde and then permeabilized with 0.5% Triton-X100. Cells were then sequentially incubated with primary antibodies (rabbit polyclonal anti-HDAg and mouse monoclonal anti-hnRNPC) for 1 h at room temperature, followed by incubation with appropriate secondary antibodies conjugated either with FITC or Texas Red (Jackson ImmunoResearch Laboratories). After washing, the coverslips were mounted in Vectashield (Vector Laboratories).

For fluorescence *in situ *hybridization a digoxigenin-11-dUTP-labelled probe was used. This probe was prepared by nick-translation of plasmid pSVL(D3) [19] as previously described [24]. This plasmid contains a trimer of full-length genomic HDV cDNA cloned in pSVL (GE Healthcare). Cells, grown on coverslips, were fixed with 3.7% formaldehyde, permeabilized with 0.5% Triton-X100, and treated with 200 U/ml RNase free DNase I (Roche) as earlier described [24]. Cells were then sequentially incubated with 70% formamide and 50% formamide at 73°C, for 3 min. Hybridization was carried out overnight at 37°C and the digoxigenin-labeled probe was detected after incubation with a monoclonal anti-digoxigenin antibody conjugated with FITC (Roche) followed by incubation with a secondary anti-FITC antibody conjugated with Alexa-488 (Jackson ImmunoResearch Laboratories).

### Confocal microscopy and image analysis

Fluorescent labeled samples were analyzed under a Zeiss LSM 510 META confocal microscope. Green fluorescence was detected using a 488-nm Argon laser and red fluorescence was detected using a 543-nm HeNe laser. The equipment was calibrated with multicolor fluorescent beads (Molecular probes, USA) and a dual-band filter allowing simultaneous visualization of green and red fluorescence.

Co-localization coefficients were calculated using ImageJ software and the JACoP plugin [25]. The Manders' overlap coefficient (OC) was calculated in order to evaluate co-localization between pairs of images in the red and green channels. The values of Mander's coefficients range from 0 to 1, with 1 indicating 100% co-localization and 0 indicating no co-localization [26].

### siRNA knockdown assays

The knockdown of endogenous hnRNPC in Huh7 cells was performed using a short hairpin RNA (shRNA) strategy.

To construct shRNA-expressing vectors, oligonucleotides targeting the human hnRNPC mRNA and the corresponding complementary sequences, were inserted into the pSIREN-RetroQ vector (BD Biosciences). The oligonucleotide sequences were designed according to the guidelines provided in http://bioinfo.clontech.com/rnaidesigner, and were as follows: 5'gatccgcagtagagatgaagaatgttcaagagacattcttcatctctactgcttttttacgcgtg 3' (sense) and 5'aattcacgagtaaaaaagcagtagagatgaagaatgtctcttgaacattcttcatctctactgcg 3' (antisense). The underlined sequences denote the hnRNPC shRNA sequence targeting the hnRNPC mRNA (nucleotides 673 to 691). The oligonucleotides were annealed *in vitro *and subcloned into the *BamH *I and *EcoR *I sites, downstream of the human U6 promotor thus yielding the pSIREN-RetroQ/hnRNPC vector. A control vector was also generated by using the negative control siRNA oligonucleotide targeting the luciferase mRNA supplied by the manufacturer (pSIREN-RetroQ/Luc).

Transfection procedures were performed using the FuGENE6 transfection reagent (Roche), according to the manufacturer's instructions. Initially, Huh7 cells were transfected with 1 μg pSVL(D3). After 48 h incubation cells were transfected again with pSIREN-RetroQ/hnRNPC or pSIREN-RetroQ/Luc. 24 h post-transfection, puromycin was added at a final concentration of 2 μg/mL. Finally, cells were harvested and analyzed by western blot as described above.

## Results

### Identification of S-HDAg interacting proteins in a yeast two hybrid screening

**Figure 1:**
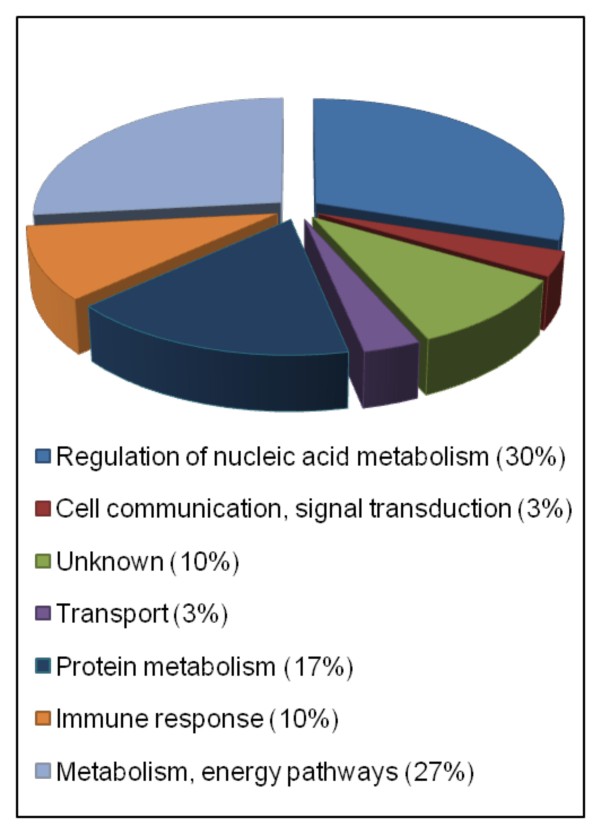
**S-HDAg interacting partners in a yeast two-hybrid screen**. The identified proteins were grouped according to their respective biological functions using the human protein reference database http://www.hprd.org/.

In order to identify proteins interacting with S-HDAg, a yeast two hybrid screening was performed. The entire S-HDAg cDNA was fused in-frame to the Gal4 DNA binding domain and the resulting construct was transformed into the yeast strain AH109. Using this bait, we screened approximately 10^8 ^clones from a human liver cDNA library, previously pre-transformed into the yeast strain Y189. Yeast colonies were first selected on medium lacking leucine, tryptophan, histidine and adenine and subsequently assayed for α-galactosidase activity. Plasmids were isolated from 112 positive clones and rescued by transformation into *E. coli*. The identity of the obtained plasmids was established by sequencing allowing the identification of 30 known proteins in the GenBank database (Table 2). These proteins were found to be involved in different metabolic pathways and functions, namely cell communication and signal transduction, transport, protein metabolism, immune response, and regulation of nucleic acid metabolism (Figure 1). In a previous study aimed to detect S-HDAg interactors, Cao et al. [8] used a combined immunoprecipitation and mass spectrometry approach. The authors reported the identification of over 100 known proteins able to interact with FLAG-S-HDAg. Among the identified proteins three were also detected as S-HDag interactors in this study: hnRNPC, hnRNP A/B, and the RNA binding protein RALY. Interestingly, these proteins are involved in the regulation of RNA metabolism and belong to the same functional group allowing to speculate about a possible involvement in HDV replication. Taking into consideration that hnRNPC was previously shown to modulate the replication of a number of RNA viruses including HCV, VSV, and influenza A [14-17],

**Table 2:** Proteins that interact with S-HDAg in the yeast two-hybrid screening

Protein	Number of clones	Biological function
Heterogeneous nuclear ribonucleoprotein C isoform 1 (HNRNPC) [NP_004491.2]	5	
	
Raly RNA binding protein-like (RALY) [NP_057951.1]	1	
	
Chromodomain helicase DNA binding protein 7 (CHD7) [NP_060250.2]	1	
	
ELAV-like protein 1 (ELAVL1) [NP_001410.2]	1	
	
Heterogeneous nuclear ribonucleoprotein A/B (HNRNPAB) [NP_112556.2]	1	Regulation of nucleic acid metabolism
	
Ankyrin repeat domain 11 (ANKRD11) [NP_037407.4]	1	
	
SWI/SNF related. matrix associated actin dependent regulator of chromatin subfamily a member 2 (SMARCA2) [NP_003061.3]	1	
	
Zinc finger proteín 533 (ZNF533) isoform 2 [NP_001106868.1]	1	
	
Ribosomal protein S15 (RPS15) [NP_001009.1]	1	

TRAF and TNF receptor associated protein [EAW55458.1]	1	Cell communication, signal transduction

Ribosome receptor P180 [BAF73807.1]	1	
	
EBNA1 binding protein 2 (EBP2) [NP_006815.2]	1	Unknown
	
Ribosomal L1 domain containing 1 (RSL1D1) [CAA07491.1]	1	

Apolipoprotein E (APOE) [NP_000032.1]	5	Transport

Proteasome activator complex subunit 2 (PSME2) [NP_002809.2]	1	
	
BRCA1 associated protein (BAP1) [NP_004647]	1	
	
Antithrombin III (SERPINC1) [NP_000479.1]	1	Protein metabolism
	
Ribosomal protein L36A like (RPL36AL) [NP_000992.1]	1	
	
Ribosomal protein L13 (RPL13) [NP_000968.2]	2	

α-1 acid glycoprotein (ORM1) [NP_000598.2]	5	
	
Complement component C3 (C3) [NP_000055.2]	1	Immune response
	
Natural killer tumor recognition sequence (NKTR) [NP_005376]	1	

17β hidroxyesteroid dehydrogenase 4 (HSD17B4) [NP_000405.1]	2	
	
17β hidroxyesteroid dehydrogenase 6 (HSD17B6) [NP_003716.2]	1	
	
ATP synthase subunit 8 [ACQ75157.1]	2	
	
Cytochrome oxidase subunit 3 (MT-COIII) [ACT53100.1]	1	Metabolism, energy pathways
	
NADH dehydrogenase subunit 2 (MT-ND2) [ACT53095.1]	1	
	
NADH dehydrogenase subunit 4 (MT-ND4) [ACQ76111.1]	1	
	
NADH dehydrogenase (ubiquinone) 1β subcomplex subunit 7 (NDUFB7) [NP_004137.2]	1	
	
**Figure 2:**
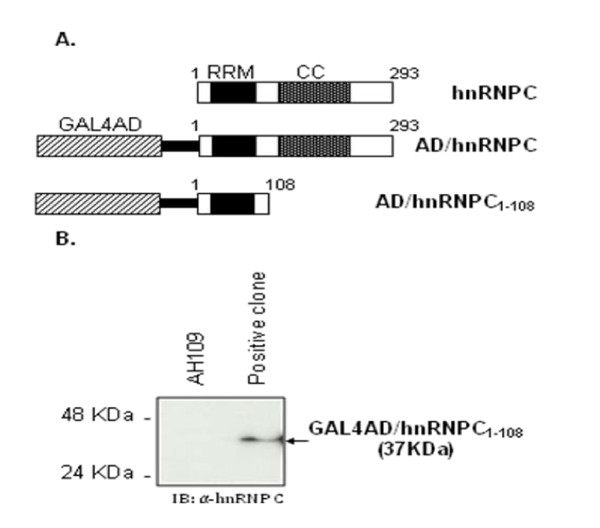
**Identification of hnRNPC as an S-HDAg interactor in a yeast two hybrid screening**. (A) Schematic representation of hnRNPC and the GAL4AD fusion proteins identified in yeast positive clones (GAL4AD/hnRNPC and GAL4AD/hnRNPC_1-108_). GAL4AD/hnRNPC and GAL4AD/hnRNPC_1-108 _were identified in four and one positive clones, respectively. GAL4AD/hnRNPC_1-108 _codes for a truncated form of hnRNPC, that contains only the first 108 aminoacids. Both proteins contain a 5'untranslated region of hnRNPC mRNA (black bar). GAL4AD represents the activation domain of the GAL4 transcription factor and RRM and CC represent the RNA recognition or coiled-coil motifs, respectively, present in hnRNPC. (B) Western blot analysis of hnRNPC expression in yeast positive clones. Yeast extracts were prepared, separated on 12% SDS/PAGE gels, and immunoblotted with an anti-hnRNPC antibody. The positive clone expressing GAL4AD/hnRNPC_1-108 _was used in this analysis and AH109 yeast extracts were used as negative control.

Cytochrome oxidase subunit 1 (MT-COI) [ACI04331.1]	1	

we decided to further explore the possible interaction between S-HDAg and hnRNPC. Four of the prey plasmids from the positive clones initially obtained in the yeast two-hybrid screening contained the entire hnRNPC ORF sequence while a fifth plasmid expressed a truncated form of the hnRNPC protein (GAL4AD/hnRNPC_1-108_), as shown in Figure 2A.

Our first approach was designed in order to confirm the expression of a GAL4AD/hnRNPC fusion protein by western blot analysis. Yeast protein extracts from a positive clone were prepared and the presence of protein GAL4AD/hnRNPC_1-108 _(37 KDa) was investigated using an antibody specific for hnRNPC protein. As shown in Figure 2B, a band of the expected molecular weight could be detected in the positive clone extracts but not in yeast AH109 extracts (negative control), confirming the expression of GAL4AD/hnRNPC_1-108 _protein.

### hnRNPC and S-HDAg interact *in vitro *and *in vivo*

**Figure 3:**
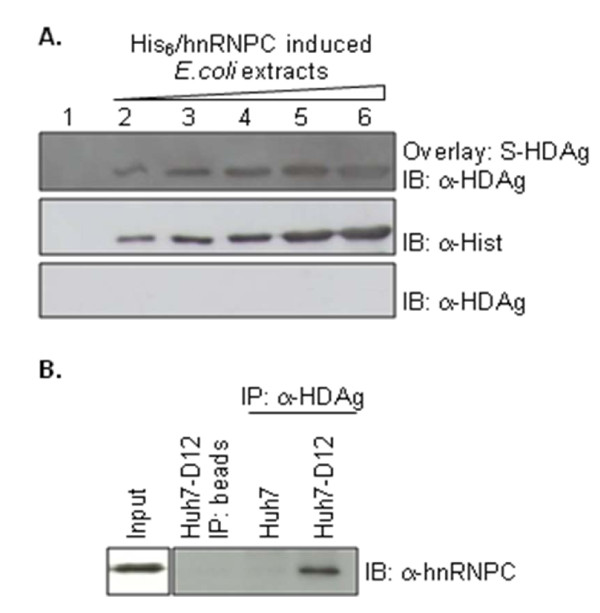
***In vitro *and *in vivo *interaction between hnRNPC and S-HDAg**. (A) Overlay of bacterially expressed His_6_/hnRNPC with recombinant S-HDAg. *E. coli *protein extracts were prepared after induction of recombinant protein expression with IPTG. Increasing amounts of His_6_/hnRNPC-containing extracts (lanes 2 to 6), were separated by SDS/PAGE, blotted onto nitrocellulose membranes and overlaid with the same amount of purified recombinant S-HDAg. Detection of S-HDAg was performed with a specific rabbit polyclonal antibody. *E. coli *protein extracts lacking His_6_/hnRNPC (lane 1) were used as a negative control (upper panel). The presence of His_6_/hnRNPC in the extracts was confirmed using a mouse anti-His-tag monoclonal antibody (middle panel). In the lower panel, the assay was performed in the absence of purified S-HDAg showing that the anti-HDAg polyclonal antibody does not recognize hnRNPC. (B) Co-immunoprecipitation of hnRNPC with S-HDAg. Huh7-D12 cell lysates were immunoprecipitated with an anti-HDAg antibody bound to protein G beads. The immunoprecipitates were separated on 12% SDS/PAGE gels and immunoblotted with an anti-hnRNPC antibody. The negative controls were performed by incubation of Huh7-D12 lysates with beads protein G beads and by immunoprecipitating Huh7 lysates with an anti-HDAg antibody.

Having established that S-HDAg and hnRNPC interact in the yeast two-hybrid assay, we decided to investigate if the two proteins also interact *in vitro *and *in vivo *in human hepatoma cells. To test for possible *in vitro *interactions we performed a blot overlay assay. To do this, both S-HDAg and hnRNPC were expressed in *E. coli*, as GST or His-tag fusion proteins, respectively. Increasing amounts of *E. coli *protein samples containing His_6_/hnRNPC were then separated by electrophoresis, and transferred to a nitrocellulose membrane. The membrane was then overlaid with recombinant S-HDAg and the bound protein was detected with a rabbit anti-S-HDAg antibody. The obtained results are displayed on Figure 3A, and show that increasing amounts of His_6_-hnRNPC correspond to a proportional increase in the amounts of bound S-HDAg, suggesting a specific interaction between the two proteins.

Having established that S-HDAg and hnRNPC interact *in vitro*, we next decided to investigate if both proteins also interact *in vivo *in the human hepatoma cell line Huh7-D12 which is stably transfected with HDV cDNA and constitutively expresses virus RNAs and antigens. This was performed using co-immunoprecipitation assays using lysates from either Huh7-D12 or the parental, non-transfected Huh7 cell lines. A rabbit polyclonal anti-HDAg antibody was used in the assays and interacting proteins were detected by western blot analysis with a mouse monoclonal antibody against hnRNPC. As negative controls we used both Huh7-D12 lysates in the absence of antibody and Huh7 lysates immunoprecipitated with anti-HDAg antibody. As shown in the Figure 3B, hnRNPC and HDAg can be co-immunoprecipitated in Huh7-D12 cells, but not in the negative controls, providing evidence that the two proteins are probably present in the same complex. It is thus possible that the two proteins also interact *in vivo *in human liver cells.

### Subcellular localization of hnRNPC, HDAg, and HDV RNA

hnRNPC proteins are predominantly localized in the nucleus and bind to nascent RNA polymerase II transcripts, playing important roles in mRNA biogenesis [27]. Since HDV viral transcription takes place in the nucleus and the involvement of RNA polymerase II in this process has been proposed [5], it is plausible to consider that hnRNPC might influence HDV replication.

**Figure 4:**
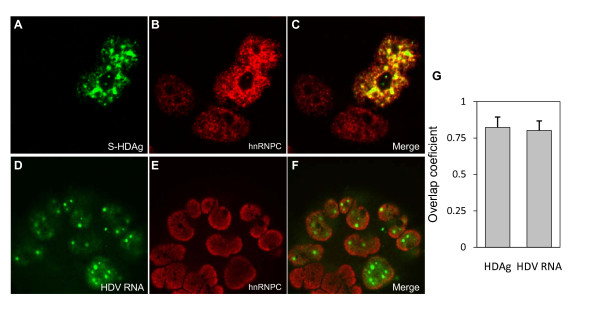
**Co-localization of hnRNPC with HDV antigens and RNA in human liver cells**. (A-C) Double indirect immunofluorescence was used to detect HDAg and hnRNPC proteins in cultured Huh7-D12 cells. Cells were fixed with 3.7% formaldehyde, permeabilized with 0.5% Triton X-100, and incubated with an anti-HDAg antibody (A, green staining) and an anti-hnRNPC antibody (B, red staining). Combined *in situ *hybridization and immunofluorescence was performed to detect HDV RNA and the hnRNPC protein. After fixation and permeabilization, cells were hybridized with a digoxigenin-labeled probe to detect HDV RNA (D, green staining) and with an anti-hnRNPC antibody (E, red staining). Overlaps of images are shown in panels C and F. (G) 10 individual cells from each experiment were analyzed using the ImageJ software and the JaCoP plugin to determine the Mander's overlap coefficient.

To learn more about the interaction between S-HDAg and hnRNPC at the cellular level, the sub-cellular distribution of the two interacting proteins was determined by double immunofluorescence staining and confocal microscopy. Huh7-D12 cells were dual labeled using FITC or Texas Red-conjugated antibodies to detect HDAg and hnRNPC respectively. The distribution of S-HDAg is consistent with previous findings [24], showing a nuclear localization, with preferential accumulation in nuclear foci (Figure 4A). hnRNPC is widely distributed throughout the nucleus and apparently overlaps with HDAg (Figure 4B). The degree of co-localization was quantified by determining Mander's overlap coefficient (OC). The mean OC (n = 10) determined for HDAg and hnRNPC was 0.823 (Std. deviation = 0.007) which indicate that the two proteins significantly co-localize. Since the diffused nucleoplasmic distribution with additional accumulation in foci of HDAg, is consistent with ongoing HDV RNA replication, the co-localization with hnRNPC may represent a biologically relevant observation.

After obtaining evidences on HDAg and hnRNPC co-localization in HDV expressing cells, we combined *in situ *hybridization and immunofluorescence experiments to analyze the co-localization between HDV RNAs and hnRNPC protein. Indeed, because HDAgs and HDV RNAs exhibit a similar sub-cellular distribution in cells undergoing HDV replication [24] and hnRNPC is also an RNA binding protein, it is likely that HDV RNAs and hnRNPC cellular distributions are also related. To exclude a possible contribution of hybridization signals corresponding to the HDV cDNA integrated in the chromosome, Huh7-D12 cells were treated with DNase prior to hybridization as earlier described [24]. Figure 4 (D-F) shows examples of the obtained results and the respective OC. The mean OC value was found to be high (0.801; std. deviation = 0.066; n = 10), indicating that both HDV RNAs and antigens significantly colocalize with hnRNPC in human cells. The observed colocalization of hnRNPC with HDV RNA and HDAgs is not inconsistent with a possible interaction during virus replication. However, this hypothesis, as well as an eventual role of the virus RNA in mediating and promoting this process, needs to be further investigated.

### Analysis of interacting domains of S-HDAg

**Figure 5:**
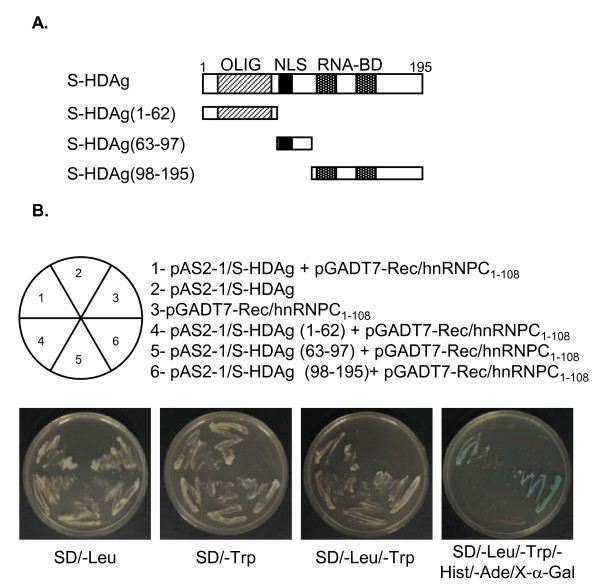
**Identification of S-HDAg domains involved in the S-HDAg/hnRNPC interaction, by yeast two-hybrid analyses**. (A) Schematic representation of the deletion constructs of S-HDAg cloned in the bait vector pAS2-1. OLIG, NLS and RNA-BD represent the oligomerization, nuclear localization signal or RNA binding domains, respectively, identified in S-HDAg. (B) AH109 yeast cells were (co)transformed with the indicated plasmids. Interactions were tested by growth selection on dropout media lacking the amino acids leucine or tryptophan (SD/-Leu or SD/-Trp) to select for pGADT7/Rec or/and pAS2-1 plasmid constructs, respectively. Activation of reporter genes was tested on plates lacking leucine, tryptophan, histidine and adenine, and containing X-α-Gal (SD/-Trp/-Leu/-Hist/-Ade/X-α-Gal).

In the yeast two-hybrid screening, we found that the N terminal aminoacid sequence of hnRNPC (amino acids 1 to 108), which includes the RNA recognition motif, was able to interact with the S-HDAg. In order to identify the regions of the S-HDAg required for interaction with this region of hnRNPC, three truncated mutants of S-HDAg were cloned fused to GAL4-BD and their interaction with hnRNPC_1-108 _was assessed using yeast two hybrid assays. Since three functional domains were previously identified in the S-HDAg, a nuclear localization signal (aa 66-75) [28], an oligomerization domain (aa 12-60) [29], and two bipartite RNA-binding domains (aa 97-143) [30], the three deletion constructs were generated in order to independently include one of the three S-HDAg functional domains. Accordingly, these constructs included the coding sequence for aminoacids 1-62, 63-97, and 98-195, respectively, in the S-HDAg (Figure 5A).

Reporter expression was reproduced using intact S-HDAg and hnRNPC_1-108_, confirming the interaction previously detected in the yeast two hybrid screening. Additionally, the C-terminal deletion mutant, containing residues 98-195, could activate the expression of reporter genes. In contrast, the two remaining constructs corresponding to aa 1-62 and 63-97, were not able to interact with hnRNPC_1-108 _and thus the activation of reporter genes could not be detected (Figure 5B). This result suggests that the S-HDAg C-terminal region containing the RNA-binding domain mediates the interaction with hnRNPC.

### hnRNPC knockdown reduces the HDAg expression levels

**Figure 6:**
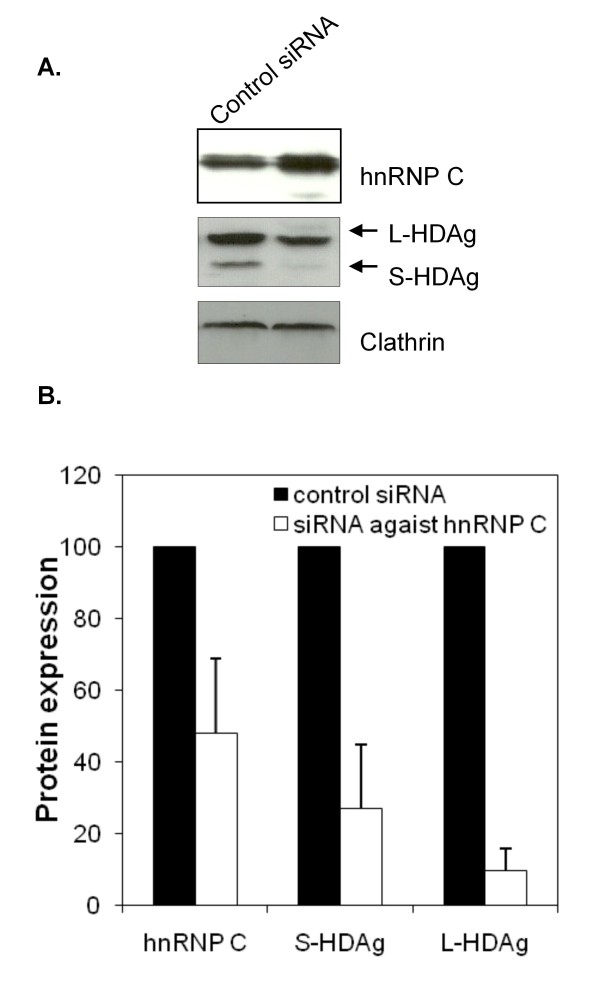
**Silencing of endogenous hnRNPC reduces expression of HDAgs**. (A) Western blot analysis of Huh7 cells transfected with plasmid pSVD3, and pSIREN-RetroQ constructs. Knockdown of the hnRNPC protein was performed using a specific siRNA in Huh7 cells. 48 hrs after addition of puromycin, total protein extracts were prepared, separated by SDS-PAGE, and transferred to a nitrocellulose membrane. Blots were incubated with anti-hnRNPC, anti-clathrin or anti-HDAg antibodies. The displayed images are representative of three independent experiments. (B) Images were digitalized and band intensity was determined using the ImageJ program. The relative amounts of protein were obtained after normalization with the housekeeping protein clathrin. Inhibition of HDAg expression is presented as a percentage after comparison with the values obtained with the negative control vector pSIREN-RetroQ/Luc provided by the manufacturer.

Several members of the hnRNP family of proteins have been recently shown to positively regulate the replication of hepatitis C and influenza A viruses [15,30]. Similarly to these two RNA viruses it would be possible that hnRNPs are also involved in the modulation of HDV replication. We thus decided to investigate the ability of hnRNPC to influence HDV replication, using a shRNA-mediated knockdown technology. Plasmid pSVL-D3, which contains three copies in tandem of the HDV genome and was previously shown to be able to initiate HDV replication [19], was used to transfected Huh7 cells. After 48 hrs incubation, the cells were transfected again with the pSIREN-RetroQ/hnRNPC vector, encoding a shRNA specific for hnRNPC, or the pSIREN-RetroQ/Luc, targeting the luciferase protein, here used as a negative control. The subsequent addition of puromycin allowed the selection of pSIREN-RetroQ expressing cells. The efficiency of the pSIREN-RetroQ/hnRNPC construct in silencing hnRNPC was analysed by western blot. Figure 6 displays the obtained results showing that in the presence of the pSIREN-RetroQ/hnRNPC plasmid, the endogenous hnRNPC levels were reduced to approximately 45% when compared with pSIREN-RetroQ/Luc transfected cells. Although the obtained reduction in intracellular hnRNPC levels was about 55%, we believe it represented an acceptable compromise in order to reduce of possible side effects in cellular metabolism. In fact, although longer incubation times resulted in a more marked inhibition of hnRNPC expression (up to 80%), cell viability was also dramatically reduced (often less than 10%) limiting the significance of the obtained results (data not shown).

After confirming that pSIREN-RetroQ/hnRNPC transfection results in a reduction of endogenous expression of hnRNPC, we decided to use the same approach to investigate the influence of hnRNPC silencing on HDAgs expression. Figure 6A shows the results obtained after western blot analysis of the relative expression of HDAgs in these experiments. As can be observed, knockdown of hnRNPC resulted in a decrease of 66% and 92% of S-HDAg and L-HDAg expression levels, respectively, when compared with the control pSIREN-RetroQ/Luc transfected cells (Figure 6B). These results suggest that hnRNPC may be involved in the modulation of HDV RNA transcription or stabilization of HDAgs coding mRNAs.

## Discussion

Despite being a small and simple virus the host factors involved in HDV replication remain largely unknown. Nevertheless, using different molecular biology and biochemical approaches a number of host proteins that interact with the virus RNA and antigens were already identified [31]. In this work we made use of a yeast two-hybrid approach to screen 10^8 ^clones from a human liver cDNA library and identify possible S-HDAg interacting proteins. The use of a similar approach has been previously reported by Wang et al allowing identifying NES1 as a L-HDAg interactor [32]. Interestingly, we found in our system that when using L-HDAg as bait this protein could self activate transcription of HIS3, ADE2, and MEL-1 reporter genes (data not shown). Accordingly, this observation did not allow us to further use L-HDAg in the yeast two-hybrid screening. However, using S-HDAg as bait, we initially obtained 112 clones that after subsequent sequence analysis and database search allowed identifying 30 known proteins (Table 2). These proteins were reported to be involved in different cellular processes including nucleic acid metabolism, cell communication, transport, protein metabolism, immune response, and energy pathways. Two of the identified proteins were found to belong to the hnRNP family of proteins: hnRNP A/B and hnRNPC. These two proteins together with the RNA binding protein RALY, also identified in this yeast two-hybrid screen, were also previously identified by Cao et al. [8] as S-HDAg interactors using an immunoprecipitation followed by mass spectrometry approach. These proteins are involved in the regulation of RNA metabolism and may thus be involved in the modulation of HDV RNA transcription and replication. This observation, and the fact that five of the positive clones detected in the yeast two-hybrid screening were found to code for hnRNPC, prompted us to further investigate a possible functional significance of the interaction between this protein and S-HDAg.

The family of hnRNP proteins has been associated with various cellular functions namely mRNA biogenesis, transport and translation [33]. Since HDV is an RNA virus highly dependent on host proteins, the identification of hnRNPC as an interactor for S-HDAg may suggest a possible role for this protein in the HDV replication cycle. Thus we decided to explore this interaction in order to elucidate its biological relevance.

Two isoforms of hnRNPC were previously identified: the more abundant hnRNPC1, and a splicing variant with a 13 aminoacid insertion, known as hnRNPC2 [12]. Both proteins form stable heterotetramers able to bind to mRNA cooperatively [13]. Although the two isoforms share most of the aminoacid sequence we were only able to find hnRNPC1 among the putative interacting clones in the yeast two hybrid screen. This result may be explained by the fact that hnRNPC2 is expressed at approximately one-third of the level of hnRNPC1 [12], thus being less abundant in the human liver cDNA library. However, the fact that we also identified a truncated form of hnRNPC comprising the first 108 amino acids which are common to both isoforms, may suggest that S-HDAg shall be able to interact with hnRNPC2 as well. A detailed analysis of the sequences obtained from all the five prey plasmids coding for hnRNPC showed an in-frame stop codon between the GAL4-AD and the hnRNPC ORF. Accordingly, the GAL4AD/hnRNPC fusion protein could only be produced through a stop codon bypass event. However it has been proposed that *S. cerevisiae *enables the synthesis of polypeptides via stop-codon readthrough [34]. Furthermore, in previous yeast two hybrid screens this mechanism was also observed, namely in the cDNA library screen with the Lyssavirus phosphoprotein as bait, where the protein dynein LC8 was identified as an interactor and the corresponding sequence also exhibited an in-frame stop codon between the GAL4-AD and the dynein LC8 coding region [35].

Having identified hnRNPC as an S-HDAg interactor in the yeast two-hybrid system, we decided to confirm this interaction *in vitro *using a blot overlay assay with purified S-HDAg and increasing amounts of bacterially expressed hnRNPC. The results indicated that the interaction of the two proteins is specific since the obtained band intensity was proportional to the amount of hnRNPC present in the bacterial protein samples. Next, we decided to investigate if S-HDAg and hnRNPC interact *in vivo *in human liver cells. This was performed using a co-immunoprecipitation assay and a polyclonal antibody against HDAg allowing confirming the previously detected interaction between the two proteins.

HDV replication occurs in the nucleus of infected liver cells. It was previously demonstrated that both virus RNA and antigens co-localize in the nucleus displaying a diffuse pattern with additional accumulation in foci. These foci, however, do not correspond to preferential sites of RNA synthesis or processing [23]. If hnRNPC is involved in HDV replication it can be expected to co-localize with HDV components in the nucleus, namely in the nucleoplasm. Immunofluoresecence and *in situ *hybridization analysis of a human liver cell line that constitutively expresses HDV RNAs and antigens, allowed calculating the Mander's overlap coefficient between hnRNPC, HDV RNA, and HDAgs. The obtained values, 0.823 for hnRNPC and HDV RNA and 0.801 for hnRNPC and HDAgs, revealed a significant degree of co-localization, supporting an eventual role of hnRNPC in virus replication.

In an attempt to map the domains of the S-HDAg that interact with hnRNPC we made three plasmid constructs coding for aminoacids 12-60, 66-75, and 98-195. These regions in the S-HDAg include the oligomerization domain, the nuclear localization signal, and the RNA binding domain, respectively. The ability to interact with hnRNPC was tested in the yeast two-hybrid assay showing that only the polypeptide comprising aminoacids 98-195 could activate transcription of reporter genes. Since in the yeast two-hybrid screen, we found that the N terminal aminoacid sequence of hnRNPC (amino acids 1 to 108), which comprises the RNA recognition motif [13] interacts with the S-HDAg it is plausible that the interaction between the two proteins is mediated by their respective RNA binding domains. However, our results seem to indicate that the interaction between the two proteins is not dependent on the presence of HDV RNA. In fact, the interaction between the two proteins can be detected in the absence of HDV RNA. Since hnRNPC is the most abundant protein in the hnRNP complexes it would be possible that the interaction with HDAg represents an artificial observation mediated by this abundance. However, taken together, our data, including blot overlay, coimmunoprecipitation and experiments aimed at mapping the interaction domains, show that the two proteins interact specifically.

hnRNPC has been shown to play important roles in the replication cycle of several RNA viruses including influenza A, hepatitis C, and polioviruses. Recently, Brunner et al, showed that the rate of poliovirus RNA synthesis is slower in a cell line expressing reduced levels of hnRNPC [36]. Furthermore, knockdown of hnRNPC using shRNAs resulted in a decrease of positive strand poliovirus RNA accumulation [37]. In this study we reduced hnRNPC expression using specific shRNAs and observed a marked decrease in both S-HDAg and L-HDAg expression suggesting a possible role of this protein in HDV mRNA synthesis, stabilization or transport. In this study we didn't investigate a possible direct effect of hnRNPC knockdown on HDV genomic or antigenomic RNA accumulation. Since S-HDAg promotes HDV RNA accumulation and L-HDAg may act as a dominant negative inhibitor of replication [38,39], reducing the intracellular amounts of both delta antigens would certainly affect virus RNA accumulation. However, this eventual effect could be directly assigned to the reduced levels of hnRNPC or represent a consequence of the diminished amounts and relative imbalance of delta antigens.

Using a combined immunoprecipitation and mass spectrometry approach hnRNPC was previously identified as one of the proteins that interact with a FLAG-HDAg fusion protein [8]. The authors performed shRNA experiments to knock down several of the identified proteins and determined the effect on the accumulation of HDV RNA. However, hnRNPC was not among the investigated proteins.

## Conclusions

In conclusion, a yeast two-hybrid screening of a human liver cDNA library allowed the identification of 30 S-HDAg interacting proteins. Among the proteins involved in nucleic acid metabolism hnRNPC was found to bind *in vitro *and *in vivo *to S-HDAg. Although shRNA-mediated knockdown of hnRNPC resulted in a marked decrease of HDAg accumulation, further studies are mandatory in order to clarify the precise role of this protein in the HDV replication cycle.

## Competing interests

The authors declare that they have no competing interests.

## Authors' contributions

AC carried out the experiments and helped draft the manuscript. MF participated in the yeast two-hybrid screening, coordination of the study and helped to draft the manuscript; ECS participated in the design and coordination of the study. CC conceived the study, participated in its design and coordination, and drafted the manuscript. All authors read and approved the final manuscript.
